# Simultaneous small bowel and colon obstruction due to splenosis. A case report and review of literature

**DOI:** 10.1016/j.ijscr.2019.03.040

**Published:** 2019-03-30

**Authors:** Alaa El-Kheir, M. Abdelnour, Jihad G. Boutros

**Affiliations:** a Department of General and Digestive Surgery, Nini Hospital, Tripoli, Lebanon; b Department of Gastroenterology and Hepatology, Maritime Hospital, Jbeil, Lebanon; c Department of General and Digestive Surgery, Maritime Hospital, Jbeil, Lebanon

**Keywords:** Splenosis, Obstipation, Intestinal obstruction, Splenic nodules

## Abstract

**Introduction:**

Splenosis, an autotransplantation of splenic tissues in another compartment of the body, is only symptomatic when hematologic diseases have recurred or compression of other adjacent structures have occurred. Therefore, we report a clinical case of a patient with bowel obstruction due to splenosis.

**Case presentation:**

A patient with a history of splenectomy, presented to the emergency room for abdominal pain and obstipation, suggesting intestinal obstruction which is also seen on CT scan. During laparotomy, multiple splenic nodules were seen on the mesentery of the small bowel and colon, with vascular bridges between them, resulting in bowel obstruction. Resection of splenic nodules was performed. The post-operative course was uneventful and the patient was discharged a week after.

**Discussion:**

Although very rare, gastrointestinal symptoms may sometimes be described in patients with splenosis. Bleeding or obstruction can occur because of intramural grow or external compression, respectively. Treatment consists of resection of these nodules.

**Conclusion:**

Splenosis should always be thought in patients with previous splenic rupture and presenting with incidental nodules seen on imaging, with further investigations when symptoms are present.

## Highlights

• Splenosis is an autotransplantation of splenic tissues throughout the body post splenic rupture or splenectomy.
• Symptomatic when hematological diseases have recurred.
• Abdominal splenosis may rarely be associated with abdominal pain and/or gastrointestinal symptoms.
• Further evaluation and treatment is mandatory when bowel obstruction is present.
• Treatment consists of resection of problematic splenic nodules.

## 1 Introduction

Splenosis is a benign acquired condition defined as an autotransplantation of splenic tissues in another compartment of the body post splenic rupture, usually due to trauma or iatrogenic (surgery) [1,2]. Knowledge of this condition is important when evaluating a patient since it is often misdiagnosed as a tumor [3]. Most cases are asymptomatic and are incidentally found during imaging studies or surgical procedures done for other conditions [4,5].

Splenosis should be differentiated from the congenital accessory spleen, which arises from the left side of the dorsal mesogastrium during the embryological period of development [4].

We present in this report a case of splenosis post splenectomy, with vascular bridges between multiple splenic nodules causing simultaneous small bowel and colon obstruction.

This report is reported in line with the SCARE criteria [6].

## 2 Case presentation

**Fig. 1:**
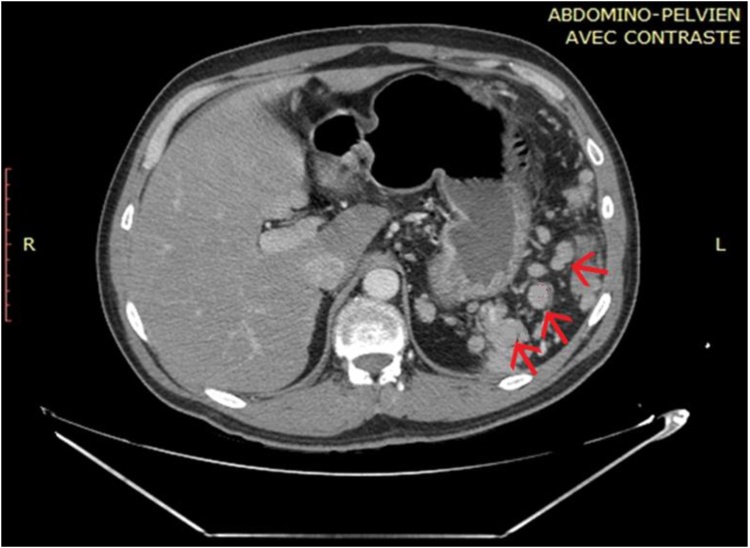
CT scan showing several splenic nodules in left upper quadrant and on the mesentery of the bowels (red arrows).

**Fig. 2:**
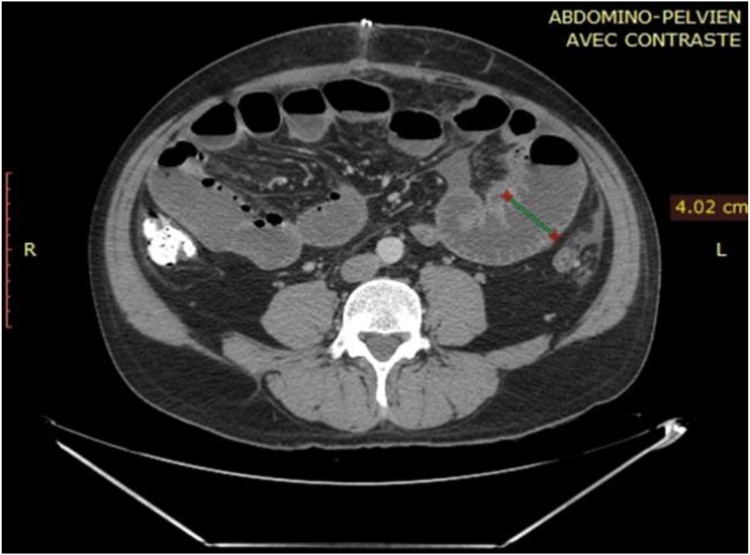
CT scan showing small bowel dilatation of 4.02 cm (green line).

**Fig. 3:**
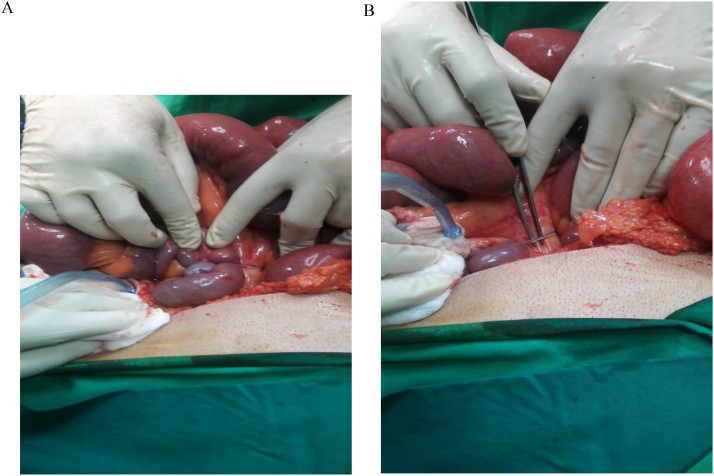
a) and b: Intra-operative images of splenic nodules and bridges on the bowels and their mesentery.

**Fig. 4:**
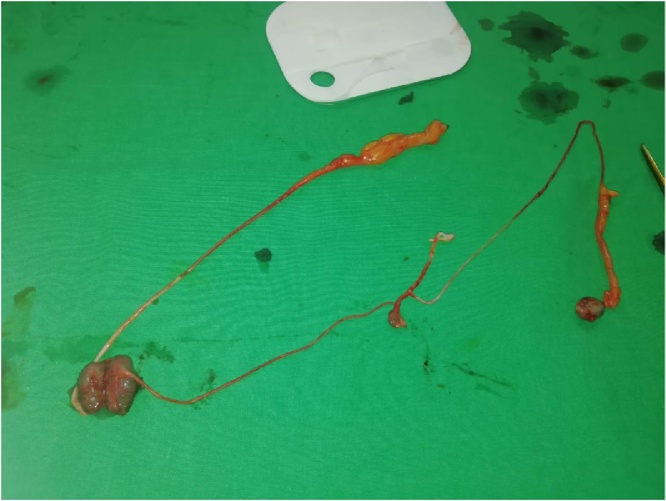
Image of the three splenic nodules and their vascular bridges after resection.

A 46 year old male patient presented to our emergency department with a 24 h history of diffuse abdominal pain and obstipation. It was not associated with vomiting or fever. The patient has a negative medical history, but a surgical history of splenectomy 12 years ago, post traumatic rupture of the spleen. On presentation, patient was afebrile, and vital signs were within normal limits. On physical exam, his abdomen was distended with diffuse tenderness. His blood tests showed a high White blood cell count of 23040/ mm³[4000–10500], with normal hepatic and pancreatic enzymes. CT scan of abdomen and pelvis showed multiple splenic nodules in the left upper quadrant, with small bowel distention and air-fluid levels mostly in the jejunum, suggesting an intestinal obstruction. A decision for exploratory laparotomy was made Fig. 1, Fig. 2. At exploration, more than 50 splenic nodules were found in the left sub-diaphragmatic region, not affecting adjacent structures. However, a splenic tissue was found on the mesentery of the jejunum, taking its vascularization from thesplenic artery and vein. This splenic tissue was giving another splenic nodule on the ileum its blood supply, which in turn is giving another splenule on the mesosigmoid its vascularization. The bridges between these splenic fragments were causing an external compression and obstruction of the small bowel and colon Fig. 3. These three splenic tissues were resected with their blood supply and sent for pathologic studies Fig. 4, while the others in the left sub-diaphragmatic area were left intact. The patient started oral feeding on post-operative day 4 after removal of the nasogastric tube and discharged home on 7th post-operative day. Pathologic studies later on confirmed the diagnosis of splenosis.

## 3 Discussion

First described by Albrecht in 1896, the term splenosis was first used by Buchbinder and Lipkoff in 1939 [7]. Its incidence is certainly underrated because the majority of cases is asymptomatic, but reports showed that 16–67% of patients with traumatic rupture of the spleen have splenosis [8].

Differentiation between splenosis and accessory spleen is not so complicated, as accessory spleens are usually fewer in number, with a maximum of six, whereas more than 100 splenic nodules can be found in the other case [2]. Accessory spleen also has a blood supply arising from the main splenic artery, however, in splenosis, it arises from the surrounding tissues and vessels [2,9]. Finally, splenic nodules are not only found near the splenopancreatic or gastrosplenic ligaments as the accessory spleen, but anywhere in the intraperitoneal space (small intestine, mesentery, greater omentum, liver…), in the retroperitoneum, thorax and subcutaneous tissues [2,3,5,9,10].

This mechanism starts with splenic rupture, either from surgery or trauma. The splenic pulp is spilled in the surrounding structures and the seeding begins, giving birth to multiple splenic nodules over time [2,7]. Another mechanism of autotransplantation is via hematogenous spread, explaining mainly the pathophysiology of intracranial splenosis [11].

Most patients are asymptomatic, and splenosis is found during imaging or surgical procedures done for other conditions. When symptoms persist, it consists mainly of abdominal pain (maybe due to infarction), bowel obstruction, caused by external compression, and gastrointestinal bleeding due to intramural grow of splenic nodules in the bowels [2,5,6,12,13]. When thoracic splenosis is found, it is manifested by hemoptysis and pleurisy [14]. In cases of splenectomy for hematological diseases, recurrence of the disease was found, and Howell-Jolly bodies cannot be seen on peripheral blood smear, indicating functioning splenosis [6,15].

Although splenosis is a benign condition, it can mimic malignant lesions such as lymphoma, therefore diagnosis can be challenging. It is seen usually incidentally on ultrasound, CT scan or MRI, which can’t confirm the diagnosis; however, CT scan with IV contrast may lead to the correct diagnosis in a patient with previous splenectomy or splenic trauma [9,16]. MRI with IV administration of iron oxide is another new method for diagnosis of splenosis. Reticuloendothelial system, including the spleen, filters these iron oxides, rendering the contrast agent tissue specific. Ectopic splenic tissue demonstrates then the same signal intensity as the normal spleen [9,17]. The diagnostic tool of choice remains the noninvasive nuclear scintigraphy. Using heat damaged RBCs tagged with Technetium-99 has been shown to be more sensitive and specific than scintigraphy using sulfur colloid [18]. One possible reason is that the spleen takes up only about 10% of the injected sulfur colloid, while greater than 90% of damaged RBCs [19].

The diagnosis of splenosis being confirmed, no further evaluation or treatment is recommended when the patient is asymptomatic, as no death from splenosis was described in the literature. However, presence of symptoms prompts therapeutic intervention. Our case is the first case described in the literature where bridges between different splenules were causing the bowel obstruction, while in the other rare cases, it was caused by intra-mural splenic growth, adhesions or mechanical obstruction from scarring and shortening of the mesentery. The mechanism of this presentation is not totally understood, but the authors believe that it’s caused not by neovascularization, but by elongation of branches of splenic vessels after traumatic fragmentation, with splenules migrating downward to the mesentery of the bowels due to gravity.

## 4 Conclusion

Splenosis should be always kept in mind in patients with previous splenic rupture and/or splenectomy, showing incidental nodules on imaging done for other conditions, thus avoiding unnecessary invasive diagnostic procedures; while further evaluation is recommended when symptoms like bleeding and obstruction are present.

## Conflicts of interest

We have no conflict of interest to declare.

## Sources of funding

No funding source.

## Ethical approval

The submitted article is a case report, ethical approval has been exempted by our institution.

## Consent

The patient was consented for operation and for publication of the case report and imaging.

## Author contribution

Alaa El-Kheir: paper concept, design, data collection, interpretation and writing the manuscript. Jihad Boutros and M. Abdelnour: reviewing and editing. Jihad Boutros was the operating surgeon and responsible for drafting and revising the article content and for the final approval of the manuscript prior to submission.

## Registration of research studies

Not applicable.

## Guarantor

Dr. Jihad G. Boutros.

## Research registry

N/A.

## Provenance and peer review

Not commissioned, externally peer-reviewed.
